# Subdural block following epidural labor analgesia: A case report

**DOI:** 10.1097/MD.0000000000043335

**Published:** 2025-07-18

**Authors:** Yingchun Shi, Lei Cheng, Yajun Xu

**Affiliations:** a Department of Anesthesiology, Dongyang Women and Children’s Hospital, Dongyang, Zhejiang Province, China.

**Keywords:** case report, complications, epidural, labor analgesia, subdural block

## Abstract

**Rationale:**

Epidural labor analgesia is a widely accepted technique for adequate pain relief during childbirth. It has gained popularity in recent decades because it ensures good analgesia and comfort for the parturient. This technique sometimes leads to complications such as subdural blocks.

**Patient concerns:**

A 25-year-old full-term pregnant woman developed motor weakness and a sensory block to T8 20 minutes after the medication was given, followed by hypotension and sensory block to T2.

**Diagnoses:**

The woman developed a subdural block after epidural labor analgesia at the L2–L3 interspace.

**Interventions:**

Norepinephrine and atropine stabilized her vital signs, while fetal monitoring remained stable.

**Outcomes:**

With treatment, the woman delivered a healthy newborn with an Apgar score of 10. A postpartum computed tomography scan excluded subarachnoid involvement, and the catheter was removed without further complications.

**Lessons:**

The vital signs, motor block, and pain were closely monitored and provided indications for the early detection of subdural blocks in this case report. The good prognosis of this case and no complications after delivery also suggested an early detection, timely treatment, and well-set management of subdural blocks can minimize the grave outcomes of the subdural blocks.

## 1. Introduction

Epidural labor analgesia is a common method that guarantees effective pain relief for patients in labor and provides considerable advantages by enhancing maternal comfort and reducing stress for both mother and fetus.^[1]^ Generally safe and widely used, the procedure nevertheless carries some risks.^[1]^ Rare but possible complications include dural puncture, subarachnoid block, or as discussed in this paper-subdural block.^[1]^ Subdural blocks are complex in that their presentation may overlap with other types of block, and the onset of symptoms may be delayed. Timely recognition and effective management of such complications are crucial in ensuring good maternal and fetal outcomes.^[1,2]^

We herein report a case of subdural block following epidural labor analgesia and also highlight the difficulty in diagnosis, including the role of vigilant monitoring during labor analgesia. This case thus underlines the necessity for increased awareness among clinicians regarding the possibility of subdural block even with routine epidural procedures.

## 2. Case presentation

This case report was approved by the institutional ethical review board of Dongyang Maternal and Child Health Hospital (Approval No. 2024-[18]). The patient has given his written informed consent for this study and the publication of his related data.

A 25-year-old gravida at 41 weeks of gestation (height 161 cm) with no previous medical history was admitted on October 11, 2023, for labor in ASA II. Her vital signs upon admission were as follows: blood pressure (BP) 115/82 mm Hg, heart rate (HR) 99 beats/min (bpm), respiratory rate (RR) 18 breaths/min, and temperature 36.2 °C. At the time of admission, the following abnormal laboratory test results were recorded: hemoglobin 112 g/L, fibrinogen 4.17 g/L, D-dimer 1.10 µg/mL, white blood count 6.55 × 10^9^/L, and neutrophils were 78.8%. The remaining parameters were normal.

On the second day, BP was 122/72 mm Hg, HR 90 bpm, RR 18 breaths/min, cervical dilation 3 cm, fetal HR 145 bpm, and contractions lasting 30 seconds every 2 to 3 minutes. The visual analog scale pain score was 3.

**Figure 1.**
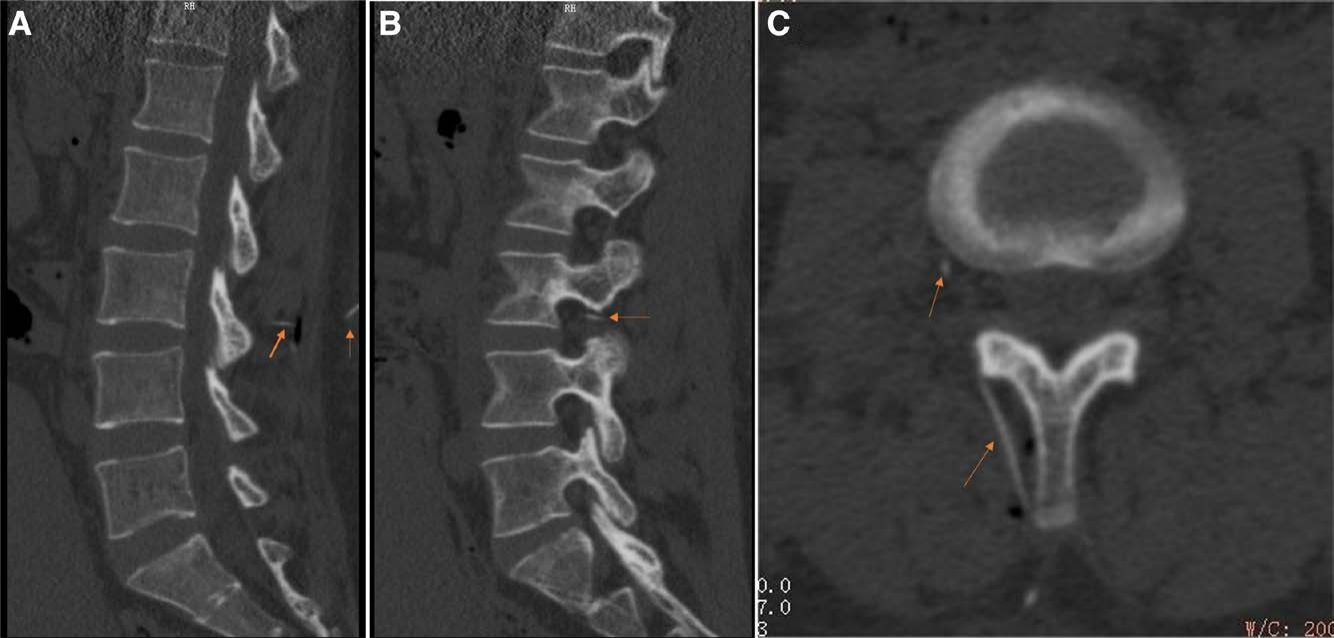
CT images. (A) Sagittal view of the CT spine demonstrating epidural catheter (arrow) that extends in via the midpoint of the L2–3 interspinous space at the vertebral level of L3. (B) Sagittal view of the CT spine showing the epidural catheter (arrow), which after its deviation from the midline is located at the right side close to the L3–4 intervertebral foramen. (C) Transverse view of the CT spine showing the epidural catheter (arrow) continues to course significantly off midline to the right posterior lower aspect of the L3 vertebral body.

For labor analgesia, a reinforced epidural catheter was inserted at the L2–3 interspace (Fig. 1A) in the left lateral decubitus position. After deviating from the midline, the dural membrane on the right foramen side of L3–4 was punctured, and it was determined from (Fig. 1B and C) that the epidural catheter horizontally circumvented the medial facet of the right articular process and reached the right posterior inferior side of the L3 vertebral body. No blood or cerebrospinal fluid was aspirated during the procedure. After a test dose of 4 mL of 1% lidocaine, a solution consisting of 10 mL of a local anesthetic was administered. The solution was prepared with 1% ropivacaine 15 mL batch no. 2K0271C71 Qilu Pharmaceutical, and sufentanil 50 µg batch no. 31A021411 Yichang Humanwell Pharmaceutical diluted in 100 mL saline. Then, an electronic patient-controlled analgesia (PCA) pump was connected: Bochuang Medical Equipment, background infusion 6 mL/h, PCA dose 5 mL, lockout interval 15 minutes. After another 20 minutes, the patient’s motor function was preserved without impairment; Bromage score: 0. The sensory block level reached T10 with a visual analog scale score of 0. After 25 minutes, with the inability to void and numbness of both legs, the Bromage score was 1, the level of sensory block was at T8. Catheterization yielded 400 mL of urine. BP was 102/83 mm Hg, HR 96 bpm, peripheral oxygen saturation (SPO_2_) 99%, fetal HR 137 bpm, and contractions were regular at 20 to 30 seconds every 3 to 4 minutes. At 30 minutes, BP dropped to 85/56 mm Hg, HR 98 bpm, and sensory function decreased to a sensory block level at T4. The PCA pump was discontinued, IV fluids increased, and nasal oxygen supplied. Fetal HR was 135 bpm, cervical dilation 8 cm, while contractions weakened, lasting 20 seconds every 3 to 4 minutes. After 35 minutes, the patient was somnolent, BP was 64/47 mm Hg, with a HR of 120 bpm, SPO_2_ of 95%, with no sensation in the lower limbs. Thus, the level of sensory block rose to T2; however, the upper limb had normal sensation and grade V muscle strength. Fetal HR varied between 90 and 132 bpm for 1 minute with contractions that lasted 20 seconds every 5 to 6 minutes (Table 1).

**Table 1:** Vital signs and labor progression following subdural block.

Parameter	20 min post-analgesia	25 min post-analgesia	30 min post-analgesia	35 min post-analgesia
Blood pressure (mm Hg)	102/83	102/83	85/56	64/47
Heart rate (bpm)	96	96	98	120
SPO_2_ (%)	99	99	N/A	95
Fetal heart rate (bpm)	137	137	135	90–132
Cervical dilation	N/A	N/A	8 cm	N/A
Uterine contractions	20–30 s every 3–4 min	20–30 s every 3–4 min	20 s every 3–4 min	20 s every 5–6 min
Sensory block level	T10	T8	T4	T2
Bromage score	0	1	N/A	N/A
VAS pain score	0	N/A	N/A	N/A
Urine output	N/A	400 mL	N/A	N/A
Motor function	Intact	Numbness in legs	N/A	No sensation in lower limbs

Bpm = beats/min, VAS = visual analog scale.

### 2.1. Diagnostic criteria

Diagnostic features for subdural block as proposed by Lubenow et al^[3]^ were fulfilled: primary criteria, negative aspiration test, and extensive sensory nerve block following the administration of drug; secondary criteria, delayed onset of sensory and motor block, more than 10 minutes, motor block despite small doses of local anesthetic and disproportionate sympathetic block considering the dose of local anesthetic administered. Diagnosis was established by meeting at least 1 primary criterion and a secondary criterion.

### 2.2. Treatment and outcomes

The patient was placed in left uterine displacement and received intravenous atropine 0.5 mg and norepinephrine 0.2 mg, which was then infused at 2 mg/h. Subsequent to vasopressor therapy, her BP improved to 98/67 mm Hg, HR to 76 bpm, SPO_2_ to 98%, RR to 18 breaths/min, fetal HR to 142 bpm, and contractions lasted 20 seconds every 5 to 6 minutes. She was then transported to the operating room for further monitoring of arterial BP. Aspiration through the epidural catheter showed no cerebrospinal fluid. After 50 minutes, BP was 116/97 mm Hg, HR 102 bpm, SPO_2_ 100%, RR 22 breaths/min, and fetal HR 122 bpm. Arterial blood gas revealed pH 7.4, partial pressure of carbon cioxide 28.7 mm Hg, and partial pressure of oxygen 129 mm Hg. Until 1.5 hours, the anesthesia level reached T4, and the vasopressor administration was gradually reduced. At 2 hours, full dilatation of the cervix had taken place, with a Bromage score of 2, and anesthesia levels receded to T8; BP was 110/67 mm Hg, HR 129 bpm, and SPO_2_ 100%. After 2.5 hours, the patient regained partial lower limb movements; her Bromage score improved with an anesthesia level till T10 and vasopressors stopped. BP was 110/69 mm Hg, HR 120 bpm, SPO_2_ 99%, and RR 18 breaths/min. At 3 hours, the patient had full lower limb movement, Bromage score was 0, and the anesthesia level at T12. BP was 110/64 mm Hg, HR 110 bpm, SPO_2_ 99%, RR 18 breaths/min, fetal HR 145 bpm, contractions were 30 seconds every 3 to 4 minutes, and the cervix was fully dilated. The Bromage score remained 0, with the anesthesia level at T12. In 3 hours 40 minutes, a healthy newborn was delivered with Apgar scores of 10–10 at 1 and 5 minutes with blood loss of 100 mL dizziness.

A CT scan performed 4 hours post-procedure showed a high-density shadow at the L3 level, consistent with the epidural catheter, which had not entered the subarachnoid space. No cerebrospinal fluid was aspirated before catheter removal. After 5 hours, the anesthetic effect had worn off, and perineal pain was present without other abnormal symptoms. An uneventful follow-up was performed without headache or dizziness after discharge. The last follow-up was in August 26, and the patient was in good condition with work and life.

Volume of drug remaining: 88 mL. Intraspinal administered anesthetics: 1% lidocaine 4 mL, 1% ropivacaine 12 mL, sufentanil 6 µg.

## 3. Discussion

### 3.1. Initial anesthetic evaluation and diagnostic criteria

In our case, the test dose of 1% lidocaine 4 mL did not indicate subarachnoid block, but the onset of the anesthetic effect, after giving 0.15% ropivacaine 10 mL (15 mg) with sufentanil 5 µg, was misinterpreted at 15 minutes as the normal epidural labor analgesia effect. The peak effect was reached at 30 minutes with a duration of 50 minutes. The symptoms of numbness and inability to move the lower extremities, with an anesthesia level reaching T2 while sparing the upper extremities, suggested characteristics like those of a subarachnoid block. Based on Lubenow criteria for subdural block, the primary criteria (negative aspiration test and extensive sensory nerve block) were met. Secondary criteria included delayed onset of sensory and motor block after more than 10 minutes, motor block with a small dose of anesthetic, and disproportionate sympathetic block.^[3,4]^ Previous report by Wills^[5]^ observed a subdural block only 2 minutes after massive subdural anesthesia in a laboring women. The slow onset of symptoms of a high-level block in our case report indicated evidence of a delayed subdural block.

CT images confirmed the deviation of the puncture needle from the midline, piercing the dura at L3–4, and the epidural catheter bypassing the right articular process to the ventral subdural space, causing both sensory and motor block. This was likely due to technical errors and anatomical abnormalities, with the patient’s tiled lumbar spinous processes complicating proper catheter placement. In this situation, 0.15% ropivacaine was used in the current case report, delivering an anesthetic effect comparable to 0.10% ropivacaine from AstraZeneca, with no motor block observed at this concentration. Similarly, ropivacaine 0.15% plus 0.5 microg/mL of sufentanil was reported to be equally effective as ropivacaine 0.10% plus 0.5 microg/mL of sufentanil for labor analgesia.^[6]^ This indicates that different drugs used in local anesthetic may not be associated with the incidence of motor block.

### 3.2. Subdural block characteristics and clinical implications

The subdural block is an unusual but potentially catastrophic complication of epidural labor analgesia. It occurs because of the accidental permeation of the local anesthetic into the subdural space between the dura mater and the arachnoid mater, which might lead to a wide extensive, high dermatomal spread of sensory and motor block of slow onset.^[4,5]^ Unlike subarachnoid blocks, which are usually immediately obvious due to the aspiration of cerebrospinal fluid in the early stages of the testing, subdural blocks are not immediately as obvious. Because of this, it would be diagnosed later or not at all in the testing. The incidence of the subdural block is <1%, hence it is an unusual but very important complication to recognize.^[7]^

### 3.3. Challenges in diagnosis and management

Overlapping symptoms with epidural or subarachnoid neural blocks often pose problems during diagnosis. Delayed onset and progressive block further complicate this clinical picture with symptoms of extensive motor and sensory block, hypotension, and sometimes respiratory involvement. The mechanisms underlying the subdural block usually consist of technical issues while placing the catheter: malposition or deviation from the path taken.^[8]^ This makes operator experience and careful monitoring during and after the administration of epidural analgesia quite important. If diagnosed early enough and managed in good time, this block will not present with serious complications such as instability in the cardiovascular system or poor fetal well-being. The case of accidental subdural block related to a cesarean delivery was reported by Elsharkawy et al,^[9]^ which found that the accident resulted in conversion disorder. This case^[9]^ underlines how challenging the diagnosis of subdural block can be and how its diagnosis requires a heightened state of clinical awareness. Our case gave a smooth born to a baby without any complications after delivery, which also emphasized that the early detection and timely management may lead to good outcomes. Walker gave a very clear guideline regarding the management of epidural analgesia in labor:^[10]^ the keys to minimizing risks rely on continuous monitoring and experience of the operator. Protocols are recommended for timely recognition and management of such complications as subdural block for keeping both mother and fetus safe.^[10]^ In this context, Toledano and Leffert go over neuraxial analgesia for labor and delivery, the timely diagnosis and treatment of less common complications like subdural block.^[8]^ They note that while the incidence is low, the potential for significant morbidity demands a high index of suspicion and suitable training for anesthesia providers.^[8]^

### 3.4. Subdural block complications

A variety of complications can arise from epidural labor analgesia, including rare instances of subdural catheter placement and related neurological effects. Accidental subdural catheterization can cause high sensory blocks, as confirmed radiologically in 1 case.^[11]^ Another report describes recurrent Horner syndrome after labor epidural, with symptoms resolving following catheter removal.^[12]^ It has also been described, though it usually settles within 24 hours, in patients receiving epidural analgesia with diluted bupivacaine.^[13]^ Subdural block during labor epidural was found in a parturient who had undergone corrective surgery for scoliosis. It was managed through reduction of anesthetic drug dose and close observation.^[14]^ Conversion disorders may complicate subdural blocks in some instances and can render their diagnosis difficult in labor as well as during cesarean delivery.^[9]^ Accidental subdural catheter placement can also lead to unexpected upper limb sensory blocks,^[15]^ while test doses of lidocaine sometimes may not identify the subdural placement, with subsequent wide extent neural blockade.^[16]^ Finally, spontaneous resolution of symptoms from a subdural block was observed in a multipara, after which analgesia was successfully established following catheter replacement.^[17]^ Luckily, the women in our case did not report any complications after the well-treated management after delivery.

The American Academy of Family Physicians took a wider perspective on labor analgesia and gave an overview of the following complications, among others: subdural block. Their wide-ranging guidelines emphasize that “skilled clinical practice and close monitoring of the patient are essential in minimizing the risks from epidural analgesia.”^[18]^ The authors note that subdural block is an uncommon but serious complication whose early detection and treatment can help prevent serious consequences. It may thus be difficult to diagnose on account of symptoms overlapping with other neural blocks. Vigilance and timely intervention are therefore very important. It is suggested that health practitioners are well-trained and maintain a high degree of suspicion for the block, especially when symptoms of extensive motor and sensory block, hypotension, and respiratory involvement are seen post-epidural administration.^[18]^

### 3.5. Differentiating between block types

Differentiating between subdural, epidural, and subarachnoid blocks can be challenging due to overlapping symptoms, but there are key distinctions that clinicians can use: epidural blocks may cause segmental sensory and motor block, hypotension, and urinary retention; subdural blocks can result in extensive motor and sensory block, hypotension, and sometimes respiratory involvement. The onset and duration also differ: the onset of epidural blocks is usually slower, can be titrated to effect, and the effect may be extended by additional drug injections; in subdural blocks, the onset is often delayed but the block develops progressively; whereas in the case of subarachnoid blocks, the onset is generally quicker and provides a dense block.^[19,20]^ Diagnostic techniques also vary: the loss of resistance technique is commonly used to identify the epidural space; subdural block diagnosis can be challenging due to the lack of cerebrospinal fluid aspiration and often requires imaging and clinical correlation,^[15]^ while aspiration of cerebrospinal fluid confirms the correct placement in the subarachnoid space. Complications differ as well: epidural blocks can lead to epidural hematoma or abscess if not properly managed; subdural blocks can result in inadequate pain relief and potential complications like cardiovascular instability, and subarachnoid blocks carry the risk of post-dural puncture headache and more severe complications if not managed correctly.^[20]^

### 3.6. Improvement strategies

This discussion highlights the importance of close monitoring and timely intervention related to labor analgesia. The critical points revealed in this discussion include: (a) late diagnosis and management of hypotension, (b) poor reporting of vital signs by the nursing personnel, and (c) failure to escalate the care to seniors on time. Fortunately, despite the high block, cardiac and respiratory functions were not affected. The corrective measures include smaller test doses of lidocaine (<3 mL) which would serve to estimate a possible subarachnoid block; observing the anesthetic effect closely for at least 30 minutes; setting critical monitoring values such as BP, HR, RR, SPO_2_, Bromage score, and analgesia plane; and finally, timely escalation of care in case of an adverse event.

## 4. Conclusion

This case illustrates the care that must be undertaken when performing epidural labor analgesia, especially in patients with anatomical peculiarities. The vital signs, motor block, and pain were well observed to provide the early detection of subdural blocks after analgesia. The smooth production of the baby and no complication after delivery in this case suggested that, an early detection, a timely treatment and well-set management on subdural blocks can minimize grave outcomes of the subdural blocks.

## Author contributions

**Conceptualization:** Yingchun Shi.

**Data curation:** Lei Cheng.

**Formal analysis:** Lei Cheng.

**Investigation:** Yajun Xu.

**Methodology:** Yajun Xu.

**Project administration:** Yingchun Shi.

**Supervision:** Yingchun Shi.

**Writing – original draft:** Yingchun Shi.

**Writing – review & editing:** Yingchun Shi.

## References

[1] Penuela I Isasi-Nebreda P Almeida H López M Gomez-Sanchez E Tamayo E. Epidural analgesia and its implications in the maternal health in a low parity comunity. BMC Pregnancy Childbirth. 2019;19:52. 30700256 10.1186/s12884-019-2191-0 PMC6354357

[2] Djurdjevic Svraka A Svraka D Pejic D Mrdja V. Establishment of labor epidural analgesia service and its assessment: an experience in a hospital of a middle-income country. Cureus. 2024;16:e55322. 38559507 10.7759/cureus.55322 PMC10981845

[3] Lubenow T Keh-Wong E Kristof K Ivankovich O Ivankovich AD. Inadvertent subdural injection: a complication of an epidural block. Anesth Analg. 1988;67:175–9. 3341567

[4] Kang SY Cho HS Yi J. Epidural, inadvertent subdural, and combined epidural-subdural anesthesia in lumbar spine surgery: a retrospective analysis. J Pers Med. 2024;14:486. 38793068 10.3390/jpm14050486 PMC11122072

[5] Wills JH. Rapid onset of massive subdural anesthesia. Reg Anesth Pain Med. 2005;30:299–302. 15898035 10.1016/j.rapm.2005.01.002

[6] Boselli E Debon R Duflo F Bryssine B Allaouchiche B Chassard D. Ropivacaine 0.15% plus sufentanil 0.5 µg/mL and ropivacaine 0.10% plus sufentanil 0.5 µg/mL are equivalent for patient-controlled epidural analgesia during labor. Anesth Analg. 2003;96:1173–7. 12651679 10.1213/01.ANE.0000054168.36798.4A

[7] Song J Shah A Ramachandran S. Case report: rare presentations of accidental subdural block in labor epidural anesthesia. Open J Anesthesiol. 2012;2:142–5.

[8] Toledano RD Leffert L. Neuraxial analgesia for labor and delivery (including instrumental delivery). UpToDate. 2024.

[9] Elsharkawy H Khanna AK Barsoum S. Caesarean delivery complicated by unintentional subdural block and conversion disorder. Case Rep Med. 2013;2013:751648. 24348576 10.1155/2013/751648 PMC3848061

[10] Walker J. Guideline for the management of epidural analgesia in labour. Norfolk and Norwich University Hospital. 2023.

[11] Chen SH Chiueh HY Hung CT Tsai SC Wong SY. Extensive sensory block caused by accidental subdural catheterization during epidural labor analgesia. Chang Gung Med J. 2006;29:607–11. 17302226

[12] Turbelin C Mallat J. Recurrent Horner’s syndrome following epidural analgesia for labor: a case report. Medicine (Baltimore). 2020;99:e18862. 32000389 10.1097/MD.0000000000018862 PMC7004573

[13] Friedman L Godovik G Eidelman LA. Horner’s syndrome during epidural analgesia in labor: an alarming sign or a benign phenomenon? Harefuah. 2002;141:507–9, 580. [Hebrew]. 12119762

[14] Lee YS Bundschu RH Moffat EC. Unintentional subdural block during labor epidural in a parturient with prior Harrington rod insertion for scoliosis. Case report. Reg Anesth. 1995;20:159–62. 7605765

[15] Alshoubi A Newhide D. Inadvertent subdural catheter placement: a rare complication in obstetric anesthesia. Cureus. 2022;14:e27252. 36039231 10.7759/cureus.27252 PMC9402258

[16] Crosby ET Halpern S. Failure of a lidocaine test dose to identify subdural placement of an epidural catheter. Can J Anaesth. 1989;36:445–7. 2758542 10.1007/BF03005344

[17] Bergquist A Buerger C Hatfield A Hofkamp M. A 24-year-old multiparous woman with apparent subdural epidural catheter: diagnosis and management of an uncommon obstetric anesthesia complication. Proc (Bayl Univ Med Cent). 2023;36:764–6. 37829234 10.1080/08998280.2023.2241162 PMC10566438

[18] American Academy of Family Physicians. Labor analgesia. Am Fam Physician. 2012;85:447–54. 22534222

[19] Wiederhold BD Garmon EH Peterson E. Nerve Block Anesthesia. In: StatPearls. StatPearls Publishing; 2024. https://www.ncbi.nlm.nih.gov/books/NBK431109/. 28613761

[20] Avila Hernandez AN Hendrix JM Singh P. Epidural Anesthesia. In: StatPearls. StatPearls Publishing; 2024. https://www.ncbi.nlm.nih.gov/books/NBK542219/. 31194376

